# The effectiveness of education-based interventions on iodine knowledge, attitude, behaviour, and status: a systematic review protocol

**DOI:** 10.1186/s13643-026-03236-4

**Published:** 2026-06-10

**Authors:** Jhama Malla, Benjamin Gardner, Jayne V. Woodside, Sarah C. Bath

**Affiliations:** 1 Discipline of Nutrition, Exercise, Chronobiology and Sleep, School of Bioscience, Faculty of Health and Medical Sciences, University of Surrey, Guildford, GU2 7XH UK; 2 School of Psychology, University of Surrey, Guildford, UK; 3 Centre for Public Health, School of Medicine, Dentistry and Biomedical Sciences, Queen’s University Belfast, Belfast, BT12 6BJ UK

**Keywords:** Iodine, Knowledge, Awareness, Education, Intervention effectiveness, Iodised salt, Pregnancy, Lactation, Women of reproductive age, Schoolchildren

## Abstract

**Background:**

Iodine deficiency remains a significant public health issue, further highlighted by the concern of its re-emergence in multiple countries. The health burden of iodine deficiency is high, especially in those who are vulnerable to the effects of iodine deficiency, such as pregnant women, infants, and children. Studies have shown that the public has low awareness and misconceptions regarding iodine. Increasing the understanding of the importance of iodine and its sources are crucial steps in controlling iodine deficiency in populations. This proposed systematic review aims to evaluate the effectiveness of education-based interventions and identify effective strategies that are associated with improved iodine knowledge, attitude, behaviour, and/or status, which could assist in improving iodine intake and combatting iodine deficiency.

**Methods:**

This protocol follows the Preferred Reporting Items for Systematic Review and Meta-Analysis Protocols (PRISMA-P) 2015 guidelines. The electronic databases that will be used to search for relevant studies are PubMed/MEDLINE, Web of Science, ScienceDirect, Scopus, and EMBASE, with no restriction on population and date. We will also explore Google Scholar to identify relevant studies. Two reviewers will perform the screening and data extraction, and disagreements will be solved by discussion or a third reviewer. We will synthesise the findings using meta-analyses where possible or otherwise via a narrative synthesis.

**Discussion:**

The findings of the review will help to inform and guide the development and implementation of education programmes and evidence-based health interventions aimed at preventing iodine deficiency, with potential applicability to wider public health strategies.

**Systematic review registration:**

PROSPERO CRD42025065136.

**Supplementary Information:**

The online version contains supplementary material available at 10.1186/s13643-026-03236-4.

## Background

Iodine is an essential micronutrient required to synthesise thyroid hormones thyroxine (T4) and triiodothyronine (T3), which are involved in regulating metabolism, in normal growth, and neurological development [1]. Thus, due to its critical role in brain and neurological development, pregnant women, infants, and young children are more vulnerable to the consequences of inadequate iodine [2]. Severe iodine deficiency in pregnancy can lead to abortion, stillbirth, congenital iodine deficiency disorder (historically cretinism), and higher perinatal and neonatal mortality [3]. Mild-to-moderate iodine deficiency in pregnancy has been associated with lower IQ and cognitive impairment in children [3, 4]. In adults, iodine deficiency can lead to hypothyroidism, hyperthyroidism, thyroid nodule formation, goitre, and decreased fertility, depending on the severity of the iodine deficiency [5]. In children, iodine deficiency may result in impaired cognitive development, stunted physical growth, development delays, goitre, and hypothyroidism, depending on the severity and timing of the deficiency [6, 7].

Iodine deficiency remains a significant global public health concern and is the leading cause of preventable mental impairment worldwide [2]. The main cause of iodine deficiency is insufficient dietary intake of iodine [1]. The richest sources of iodine are iodised salt, seafood, fish, cow’s milk, dairy products, eggs, fortified food (e.g. plant-based milk, bread) and seaweed [8], though some types of seaweed can contain a very high concentration of iodine and can lead to excess iodine consumption [9, 10]. The primary source of iodine varies between countries by dietary habits and fortification practices [11–13]. For instance, the primary source of iodine in the UK is cow’s milk, whereas fish is the primary source in Iceland [14]. Neither the UK nor Iceland have a mandatory universal salt iodisation (USI) programme [15]. In countries with mandatory USI, iodised salt represents the key source of iodine [11, 16].

While significant progress in reducing iodine deficiency has been globally achieved through effective public health programs, such as iodine fortification and monitoring programs [17], recent evidence of the re-emergence of iodine deficiency in several countries has become a growing concern [18–21]. The re-emergence of iodine deficiency may be attributed to several potential factors. The recent dietary shift towards an increasing adoption [22] and recommendation of plant-based diets [23], which are naturally low in iodine [24], can significantly reduce iodine intake if unfortified options are selected [25]. Additionally, a potential decrease in the usage of iodised salt [15], which has historically helped many countries successfully tackle iodine deficiency [11], and changes in farming practices that have resulted in a reduction of iodine content in dairy products [18], may have contributed to reduced iodine intake. Considering the adverse health implications of inadequate iodine in population, it is necessary to investigate effective strategies to address this growing concern.

Research has shown a lack of public awareness regarding the importance of iodine, iodised salt, and the consequences of iodine deficiency [26–28]. Additionally, misconception exists regarding iodised salt, with some individuals perceiving non-iodised salt, such as sea salt, as more natural and healthy [28], and others incorrectly perceiving these alternatives to be a better source of iodine than iodised salt [29]. Developing effective ways to target this knowledge gap could significantly contribute to improving iodine intake of the population.

Education is a key tool utilised in a preventative approach for improving public health and promoting behaviour change [30]. It empowers individuals and communities to make informed choices in adopting health practices by bridging knowledge gaps, enhancing understanding, and dispelling misconceptions [31]. However, the extent to which education-based interventions have been employed to address iodine deficiency and their effectiveness remains unclear. While existing reviews have examined a range of iodine-related strategies, including dietary approaches [32], salt iodisation [33], fortification [34], and reviewed iodine knowledge and awareness [27], there has been no systematic synthesis of the effectiveness of education-based interventions targeting iodine-related outcomes, specifically, knowledge, attitude, behaviour, and status. Furthermore, evidence has not been systematically reviewed on how these interventions can influence psychological, behavioural, and iodine status. Investigating these interventions in relation to iodine is essential to develop effective and sustainable strategies to tackle both the re-emergence of iodine deficiency and the persistent prevalence of iodine deficiency in many parts of the world, in order to prevent its detrimental health implications.

This systematic review will be the first to comprehensively synthesise the existing evidence to address this critical literature gap and investigate the effectiveness of education-based interventions in human populations, including subgroups vulnerable to iodine deficiency, such as women of reproductive age, pregnant and lactating women, and school-aged children, in improving iodine-related outcomes. Effectiveness will be assessed by comparing iodine-related outcomes between groups receiving iodine education and a comparator group, or by comparing outcomes before and after the intervention within the education group in single-group pre-post studies. The proposed systematic review will aim to answer the following questions:

1. What are the effects of iodine education-based interventions across all population groups (including subgroups that are vulnerable to iodine deficiency such as women of reproductive age, pregnant women/lactating women, and school children)?
2. What sources of variation are there in iodine education-based interventions (e.g. intervention components, behaviour change techniques, delivery methods, population characteristic, contextual factor) and to what extent are these sources of variation associated with the effectiveness of the intervention?

## Methods

This systematic review protocol follows the Preferred Reporting Items for Systematic Reviews and Meta-Analyses (PRISMA) Protocol checklist [35, 36]. Any amendments to this protocol will be documented and updated in the PROSPERO registration. The completed checklist is provided as an additional file (see Additional file 1) [36].

### Eligibility criteria

#### Population

The population of interest includes all individuals, regardless of age, gender, ethnicity, or geographical location, who have received iodine-related education-based interventions. Studies of iodine-related education-based interventions focusing solely on specific subgroups (e.g. women of reproductive age, pregnant women, children, adolescents) are also eligible for inclusion.

#### Intervention

We define 'iodine education-based interventions’ using Michie et al.’s (2011) [37] definition of ‘education interventions’, i.e. as attempts to increase knowledge or understanding of any aspect of iodine nutrition. These interventions may include, but are not limited to, information on iodine's role in health and development, its importance and dietary sources, consequences of iodine deficiency, and practical strategies for improving iodine intake (e.g. iodised salt, supplements, dietary choices). We will include any intervention that meets this definition, even if it incorporates additional intervention functions from Michie et al.’s (2011) [37] Behaviour Change Wheel, such as persuasion, incentivisation, training, enablement, modelling, or environmental restructuring. However, education-based interventions that include coercion or restriction will be excluded.

Intervention content will be coded for Behaviour Change Wheel intervention functions, and Behaviour Change Techniques, and interventions are characterised as low, medium, and high complexity based on the number and type of component and other key intervention characteristics (e.g. delivery format, repeated contacts, intensity). The review will not aim to isolate the independent effect of education in multi-component interventions, as the effects of individual components cannot be separated when they are delivered together and not evaluated independently. Instead, the educational component will be described within the context of the overall intervention.

Studies are eligible if they have an iodine-focused educational component and include at least one pre-specified outcome of interest (iodine knowledge, attitude, intention, behaviour, or status), with data reported at baseline and at least one post-intervention time point or include a comparator group.

Studies that do not have a clear, focused iodine educational component (e.g. those solely focused on provision of iodine-rich foods, iodine supplements, or iodised salt) will not be eligible. The review will have no restriction on intervention frequency, duration, intensity, delivery method, setting, provider, or theoretical basis. This is to ensure all the relevant evidence on this topic is gathered.

#### Study designs

Eligible study designs include randomised controlled trials (including cluster-randomised trials), quasi-experimental studies (including non-randomised controlled trials), and uncontrolled before-and-after (pre–post) studies.

#### Comparator(s)

For randomised controlled trials and quasi-experimental studies with control/comparator group, the comparator will be the control or minimal intervention group. The main outcomes will be measured by comparing changes or differences in data between the intervention and control/minimal groups (i.e. between-group differences).

For pre-post intervention studies without a control/comparator group, the comparator will be the baseline (pre-intervention) measurement. The main outcomes will be measured by comparing data before and after the intervention within the same group (i.e. within-group change).

#### Outcomes

The primary outcome of this review is iodine-related knowledge, reflecting the central target of education-based interventions. The secondary outcomes of this review include other psychological antecedents of iodine intake (attitude, and intention), iodine-related behaviours (e.g. use of iodised salt, use of supplement, consumption of iodine-rich food), and iodine status [e.g. urinary iodine concentration (UIC), thyroid hormone concentration, thyroglobulin concentration, goitre prevalence, and iodine concentration in breast milk]. If included studies also report additional outcomes that are deemed relevant by the authors, these will be extracted, even in cases where baseline measurement is challenging. For example, studies assessing the long-term outcomes in offspring (e.g. iodine status, cognitive ability) resulting from maternal iodine education interventions will be considered relevant for extraction even if baseline data for offspring is not available.

#### Outcome definitions

Knowledge outcomes will be defined as any quantitative or quantifiable measure of iodine nutrition-related knowledge (e.g. total knowledge scores, percentage of correct responses, or binary responses assessing correct responses). Attitude outcomes will be defined as any quantitative or quantifiable measure reflecting participant’s perception or beliefs related to iodine nutrition, such as perceived risk or perceived health benefits. Intention outcomes will be defined as any quantitative or quantifiable measure reflecting participant’s stated intention or plan to engage in iodine-related behaviours (e.g. plan to use iodised salt or consume iodine-rich food).

Behaviour outcomes will be defined as any quantitative or quantifiable measure of iodine-related behaviour including dietary practice (e.g. use of iodised salt, frequency intake of iodine-rich food or iodine-fortified food), as well as proxy indicators of behaviour such as purchasing practices or iodine concentration in household salt that are relevant to iodine intake.

Meaningful change or effectiveness in outcome will be interpreted as a statistically significant between-group differences (i.e. intervention versus control), while statistically significant within-group changes (e.g. pre-post change in single group or with control group studies) will be interpreted as indication of potential effectiveness.

For iodine-status outcomes, findings will additionally be contextualised using established population-level reference values where applicable (e.g. WHO criteria for urinary iodine status).

### Information sources

The electronic databases that will be used to identify relevant studies will be PubMed/MEDLINE, Web of Science, ScienceDirect, Scopus, and EMBASE. These databases have a broad coverage of nutrition, public health, behavioural, education-related, and multidisciplinary research. A comprehensive search strategy using relevant keywords related to iodine, educational intervention, and iodine outcomes, and Medical Subject Headings (MeSH) terms using Boolean operators (OR, AND) has been developed for PubMed/MEDLINE (see Additional file 2) and will be adapted for each database to ensure comprehensive retrieval of relevant studies. In addition to the main databases, we will perform a verification search in PsycINFO and CINAHL databases to strengthen coverage of education- and behaviour-focused interventions. Furthermore, a simplified Google Scholar search will also be conducted to capture grey literature, including studies not published in peer-reviewed journals, and the first 300 records will be examined to identify any eligible studies not captured through the database searches. Reference lists of relevant studies will be screened to identify additional studies. Searches will be conducted with no date limits but will be limited to English language and human participants. If additional information is required to assess eligibility, a maximum of two attempts will be made to request more information from the study authors. The literature search will be updated if the final analysis is not completed within three months of the initial search.

### Study records and selection process

Two reviewers will independently conduct a two-staged screening process using Rayyan software. In the first stage, titles and abstracts will be screened, and articles that appear to meet the eligibility criteria will be included in the second-stage screening. In the second stage, full text of the articles will be screened against the eligibility criteria. Articles meeting any of the exclusion criteria at any stage of the screening process will be excluded, and the reasons for exclusion will be recorded. If additional information is required to assess eligibility, a maximum of two attempts will be made to request more information from the study authors.

Disagreements between the reviewers will be resolved through discussion, or through consultation with a third reviewer if a consensus is not reached. Inter-reviewer agreement will be assessed using percentage agreement and Cohen’s kappa.

Endnote version 21 will be used for reference management and to remove duplicates. A PRISMA flow diagram will be created to document the study selection process.

### Data collection process

Data extraction will be performed by two reviewers, using a piloted data extraction form. In order to minimise extraction error, interim duplicate checking of a random 20% subset of included studies will be undertaken during the initial stages of data extraction to ensure consistency and accuracy of the extracted data. One reviewer will conduct full data extraction, while the second reviewer will independently extract a 20% random sample of studies. This approach is selected as a pragmatic quality-control check that balances methodological rigor with feasibility.

Agreement between reviewers will be assessed to evaluate the consistency and reliability of the data extraction process. Full agreement is defined as agreement on the information extracted, regardless of differences in wording, provided the extracted information would lead to the same understanding, coding, and analysis. If full agreement is achieved, this will indicate that the first reviewer’s data extraction is reliable, and the first reviewer will proceed to complete the data extraction for the remaining studies independently. If discrepancies are identified, these will be discussed to understand the reasons for disagreement, and resolved through discussion, or by consultation with a third reviewer if consensus cannot be reached. The data extraction approach will be refined and updated accordingly, and an additional random subset of studies will be subjected to duplicate data-extraction to confirm full agreement. This process will be repeated as necessary until full agreement is achieved, after which the first reviewer will complete data extraction for the remaining studies. This approach minimises extraction error while remaining feasible within the scope of the review. Where multiple publications are identified as reporting the same study, data will be extracted once from the most complete report.

The following data will be extracted from the included studies:

- Study characteristics: author(s), year of publication, country, study design
- Participants: population group (including sex), age, urban/rural location, baseline data
- Intervention characteristics: Delivery method, tools/methods used, provider profession, setting, behaviour change techniques, theoretical basis (if mentioned), etc.), duration of the intervention and follow-up periods, and any other information deemed relevant by the review authors
- Outcome: tools/methods used, relevant outcomes, main findings/results
- Implementation challenges: e.g. resource barriers, funding barriers

### Risk of bias (quality) assessment

Risk of bias will be assessed using appropriate tools (for example, the Cochrane Risk of Bias Tool for Randomised Trials (RoB 2) for randomised controlled trials) by two reviewers. One reviewer will conduct a full assessment, while the second reviewer will independently assess a 20% random sample of studies. Discrepancies will be resolved through discussion, but if consensus cannot be reached, a third reviewer will be consulted. A further 20% of samples will be assessed iteratively until agreement is reached. All studies meeting the inclusion criteria will be included in the synthesis, irrespective of their risk of bias. The strength of the body of evidence will be assessed qualitatively, based on the overall risk of bias of the included individual studies and the consistency of findings across studies.

### Data synthesis

Findings will be organised primarily by outcome, with narrative synthesis and meta-analysis (where at least two studies report comparable outcome measures and quantitative pooling is judged to be informative) within each outcome domain. Within each outcome domain, studies will be further categorised by study design (e.g. RCT, quasi-experimental, pre-post). Findings will be synthesised narratively, and tabulated where appropriate to summarise results. Relevant intervention characteristics (e.g. delivery modality and theoretical underpinning) will be described descriptively alongside outcome findings to provide contextual information. Psychological outcomes (e.g. knowledge, attitudes, intentions) are expected to be measured using a range of instruments and will therefore be grouped by outcome domain. Where appropriate, outcomes measured using different instruments will be standardised as far as possible using appropriate effect size measures. However, where standardisation is not possible or meaningful due to substantial heterogeneity in measures, findings will be synthesised narratively. Where at least two studies report the same outcome using the same, or convertible metrics, quantitative synthesis will be performed using a meta-analysis; this is consistent with previous meta-analyses in iodine research [38, 39]. However, even where the minimum criteria for meta-analysis are met, narrative synthesis will be prioritised when quantitative pooling would result in several small meta-analyses of the same outcome due to different outcome measures, therefore limiting interpretability. If a meta-analysis is feasible, statistical heterogeneity will be assessed using the *I*^2^ statistic*.* If heterogeneity is greater than 50%, a random-effects model will be used, otherwise, a fixed-effect model will be considered. Meta-analyses will be conducted in R (Rstudio). Where outcome measures are not directly comparable, results will be synthesised narratively only. Additionally, studies without a no-education control or comparator group will be narratively synthesised, unless they report comparable data suitable for separate, standalone quantitative synthesis.

For controlled studies (randomised controlled trials and quasi-experimental studies with a control or comparison group), analysis will focus on between-group differences in outcomes (i.e. differences in change or final data between intervention and control groups). For uncontrolled pre-post studies, analysis will focus on within-group changes from baseline to follow-up. Analyses and synthesis of controlled and uncontrolled studies will be conducted separately to ensure fair comparisons and thus their results will not be pooled together in the same meta-analysis.

Publication bias across studies will be assessed using funnel plots and Egger’s test for outcomes where at least 10 studies are included in the meta-analysis. For outcomes with fewer than 10 studies, funnel plots and Egger’s test will not be conducted due to limited statistical power and potential unreliability. In such cases, a qualitative discussion of the potential for publication bias will be provided, considering factors such as study characteristics.

The presence of specific behaviour change techniques in the included studies will be identified, and their association with intervention effectiveness will be investigated. Additional factors, such as other intervention characteristics and contextual factors (e.g. setting, population characteristics) will also be explored, and analysed for their influence on intervention effectiveness, either narratively or, if feasible through meta-analysis. Implementation challenges (e.g. resource barriers, funding barriers) will also be documented, if reported, and included in the narrative synthesis. Furthermore, if at least ten studies per covariate are available, meta-regression will be performed to explore the relationship between study level characteristics (e.g. between behaviour change techniques, other intervention characteristics, contextual factors) and the magnitude of intervention effects.

### Analysis of subgroups or subsets

Subgroup analyses will be conducted for groups vulnerable to iodine deficiency (e.g. pregnant/lactating women, non-pregnant/non-lactating women and schoolchildren) to explore the effectiveness of educational interventions across these subgroups, where at least two studies are available per subgroup. If meta-analysis and meta-regression is not feasible, a narrative synthesis will be provided.

## Discussion

This systematic review will provide a comprehensive synthesis of effectiveness of education-based interventions, and identify the strategies associated with improving psychological antecedents of iodine intake (knowledge, attitude, and intention), iodine-related behaviours, and iodine status across all population groups. By systematically reviewing studies across diverse populations and settings, this review will provide a comprehensive overview of how strategies utilised in educational interventions impact key outcomes in various contexts.

To our current knowledge, no systematic review has been conducted that has examined the effectiveness of education-based interventions on these outcomes of interests in any population group. This represents a significant gap in the literature. Our review will address this gap by synthesising evidence not only on intervention effectiveness but also by exploring sources of variation, including intervention characteristics, and contextual factors, that may influence intervention effectiveness. Understanding both overall effectiveness and the factors underlying improved outcomes in education interventions is critical for informing effective public health strategies aimed at reducing iodine deficiency. Our planned subgroup analyses will provide a comprehensive understanding of intervention effects in vulnerable groups, which is essential for tailoring interventions to meet the specific needs of population groups at risk of iodine deficiency.

We anticipate that the number of eligible studies may be limited, which could affect the feasibility of conducting quantitative analysis such as meta-analysis and meta-regression. While we acknowledge that meta-analyses are typically conducted with multiple studies and tend to give more robust conclusion when more studies are available, we will be adopting a pragmatic approach where meta-analysis will be conducted if at least two studies report the same outcome using comparable outcome measures and sufficient data are available to calculate effect estimates, and where quantitative pooling is considered informative for interpretation. This approach is consistent with previous iodine research, where meta-analyses have been performed with as few as two studies, due to the limited number of studies with comparable outcomes. We will acknowledge the limitations of meta-analyses conducted using small number of studies (defined as fewer than 5 studies) and will provide careful interpretation of results, placing emphasis on heterogeneity, and confidence intervals, rather than statistical significance alone. Additionally, heterogeneity in study reporting, methods, design, and outcome measurement may present challenge for data synthesis. Nevertheless, where quantitative analyses are not possible, for example, where outcome measures are not directly comparable or convertible, or where meta-analysis is not judged to be informative, even when the minimum criteria for meta-analysis are met, such as when quantitative pooling would result in several small meta-analyses of the same outcome due to different outcome measures, which would limit interpretability, a thorough narrative synthesis will be provided, ensuring a transparent and comprehensive summary of the evidence.

The findings of this systematic review will be important for educators, policymakers, and researchers in guiding the development and implementation of more effective and contextually appropriate evidence-based educational intervention programmes aimed at tackling iodine deficiency, with potential relevance to developing wider public health strategies.

## Supplementary Information

Supplementary Material 1. PRISMA-P 2015 Checklist.

Supplementary Material 2. Search strategy for PubMed/MEDLINE.

## Data Availability

Not applicable.

## Abbreviations

PRISMA: Preferred Reporting Items for Systematic Review and Meta-Analyses
PRISMA-P: Preferred Reporting Items for Systematic Review and Meta-Analysis Protocols
ROB 2: Risk of Bias 2
T4: Thyroxine
T3: Triiodothyronine
UIC: Urinary iodine concentration
USI: Universal salt iodisation
