# Supplementary material for: The effectiveness of education-based interventions on iodine knowledge, attitude, behaviour, and status: a systematic review protocol
Source: Syst Rev. 2026 Jun 10;15:226. doi: 10.1186/s13643-026-03236-4 (PMC13474484; doi:10.1186/s13643-026-03236-4)
Supplement: Supplementary file 2 [file 13643_2026_3236_MOESM2_ESM.docx]

**Search strategy for PubMed/MEDLINE:**

| **Concept** | **Keywords** | **Mesh Terms** |
| --- | --- | --- |
| Iodine | "Iodine" OR "iodide" OR "iodate" OR "iodis*" OR "iodiz*" OR “dietary iodine” OR "iodine deficiency disorder*" OR "Iodine deficiency" OR "iodine nutrition" OR "iodine requirements" OR "iodi*ed salt" | "Iodine/blood"[Mesh] OR "Iodine/deficiency"[Mesh] OR "Iodine/metabolism"[Mesh] OR "Iodine/physiology"[Mesh] OR "Iodine/urine"[Mesh] OR "Iodine/therapeutic use"[Mesh] |
|  | **AND** |  |
| Educational intervention | "education*" OR "education* program*" OR "intervention" OR "education* intervention" OR "iodine education" OR "iodine knowledge" OR "prevention programme" OR "Health Promotion" OR "nutrition* education" OR "nutrition* intervention" OR "school-based program" OR "patient education" OR "Health Education" OR "training program" OR "health literacy intervention" OR "workshops" OR "health campaign" OR "promotion of health" OR "mass media" OR "mass-media campaign" OR "community engagement" OR "increasing awareness" OR "community-based intervention" OR "community trial" OR "school-based intervention" OR "one-on-one counselling" | "early intervention, educational"[MeSH Terms] OR "Health Education"[MeSH Terms] OR "Health Promotion"[MeSH Terms] OR "Education"[MeSH Terms] OR "health knowledge, attitudes, practice"[MeSH Terms] OR "Patient Education as Topic"[MeSH Terms] OR "nutritional sciences/education"[MeSH Terms] OR "nutrition disorders/prevention and control"[MeSH Terms] |
|  | **AND** |  |
| Outcome | "knowledge" OR "attitude" OR "awareness" OR "perception*" OR "belief*" OR "intention" OR "practic*" OR "behaviour" OR “behavior” OR "opinion" OR "salt sales" OR "iodi*ed salt" OR "iodine intake" OR "iodine status" OR "urinary iodine" OR "urine iodine" OR "UIC" OR "UIE" OR "thyroid hormone*" OR "thyroid stimulating hormone*" OR "TSH" OR "T4" OR "T3" OR "supplement*" OR "breast milk concentration" OR "goiter" OR “goitre” OR "iodine rich food*" OR "dairy" OR "seafood" OR "fish" OR "iodine-containing foods" OR “fortif*” OR "behavior change" OR "behaviour change" | "Health Behavior"[Mesh] |

**PubMed/MEDLINE search:**

((("Iodine"[Title/Abstract] OR "iodide"[Title/Abstract] OR "iodate"[Title/Abstract] OR "iodis*"[Title/Abstract] OR "iodiz*"[Title/Abstract] OR "dietary iodine"[Title/Abstract] OR "iodine deficiency disorder*"[Title/Abstract] OR "Iodine deficiency"[Title/Abstract] OR "iodine nutrition"[Title/Abstract] OR "iodine requirements"[Title/Abstract] OR "iodi*ed salt"[Title/Abstract]) OR ("Iodine/blood"[Mesh] OR "Iodine/deficiency"[Mesh] OR "Iodine/metabolism"[Mesh] OR "Iodine/physiology"[Mesh] OR "Iodine/urine"[Mesh] OR "Iodine/therapeutic use"[Mesh])) AND (("education*"[Text Word] OR "education* program*"[Text Word] OR "intervention"[Text Word] OR "education* intervention"[Text Word] OR "iodine education"[Text Word] OR "iodine knowledge"[Text Word] OR "prevention programme"[Text Word] OR "Health Promotion"[Text Word] OR "nutrition* education"[Text Word] OR "nutrition* intervention"[Text Word] OR "school-based program"[Text Word] OR "patient education"[Text Word] OR "Health Education"[Text Word] OR "training program"[Text Word] OR "health literacy intervention"[Text Word] OR "workshops"[Text Word] OR "health campaign"[Text Word] OR "promotion of health"[Text Word] OR "mass media"[Text Word] OR "mass-media campaign"[Text Word] OR "community engagement"[Text Word] OR "increasing awareness"[Text Word] OR "community-based intervention"[Text Word] OR "community trial"[Text Word] OR "school-based intervention"[Text Word] OR "one-on-one counselling"[Text Word]) OR ("early intervention, educational"[MeSH Terms] OR "Health Education"[MeSH Terms] OR "Health Promotion"[MeSH Terms] OR "Education"[MeSH Terms] OR "health knowledge, attitudes, practice"[MeSH Terms] OR "Patient Education as Topic"[MeSH Terms] OR "nutritional sciences/education"[MeSH Terms] OR "nutrition disorders/prevention and control"[MeSH Terms]))) AND (("knowledge"[Text Word] OR "attitude"[Text Word] OR "awareness"[Text Word] OR "perception*"[Text Word] OR "belief*"[Text Word] OR "intention"[Text Word] OR "practic*"[Text Word] OR "behaviour"[Text Word] OR "behavior"[Text Word] OR "opinion"[Text Word] OR "salt sales"[Text Word] OR "iodi*ed salt"[Text Word] OR "iodine intake"[Text Word] OR "iodine status"[Text Word] OR "urinary iodine"[Text Word] OR "urine iodine"[Text Word] OR "UIC"[Text Word] OR "UIE"[Text Word] OR "thyroid hormone*"[Text Word] OR "thyroid stimulating hormone*"[Text Word] OR "TSH"[Text Word] OR "T4"[Text Word] OR "T3"[Text Word] OR "supplement*"[Text Word] OR "breast milk concentration"[Text Word] OR "goiter"[Text Word] OR "goitre"[Text Word] OR "iodine rich food*"[Text Word] OR "dairy"[Text Word] OR "seafood"[Text Word] OR "fish"[Text Word] OR "iodine-containing foods"[Text Word] OR "fortif*"[Text Word] OR "behavior change"[Text Word] OR "behaviour change"[Text Word]) OR ("Health Behavior"[Mesh])) AND ((humans[Filter]) AND (english[Filter]))
